# Classification of Spatiotemporal Neural Activity Patterns in Brain Imaging Data

**DOI:** 10.1038/s41598-018-26605-z

**Published:** 2018-05-29

**Authors:** Min Song, Minseok Kang, Hyeonsu Lee, Yong Jeong, Se-Bum Paik

**Affiliations:** 1 Department of Bio and Brain Engineering, KAIST, Daejeon, 34141 Republic of Korea; 2 Program of Brain and Cognitive Engineering, KAIST, Daejeon, 34141 Republic of Korea

## Abstract

Various patterns of neural activity are observed in dynamic cortical imaging data. Such patterns may reflect how neurons communicate using the underlying circuitry to perform appropriate functions; thus it is crucial to investigate the spatiotemporal characteristics of the observed neural activity patterns. In general, however, neural activities are highly nonlinear and complex, so it is a demanding job to analyze them quantitatively or to classify the patterns of observed activities in various types of imaging data. Here, we present our implementation of a novel method that successfully addresses the above issues for precise comparison and classification of neural activity patterns. Based on two-dimensional representations of the geometric structure and temporal evolution of activity patterns, our method successfully classified a number of computer-generated sample patterns created from combinations of various spatial and temporal patterns. In addition, we validated our method with voltage-sensitive dye imaging data of Alzheimer’s disease (AD) model mice. Our analysis algorithm successfully distinguished the activity data of AD mice from that of wild type with significantly higher performance than previously suggested methods. Our result provides a pragmatic solution for precise analysis of spatiotemporal patterns of neural imaging data.

## Introduction

Neural activities observed in optical imaging data often show a variety of spatiotemporal patterns of global synchronization^1^ or local propagation^2–7^. A number of studies have suggested that such activities exhibit spatiotemporally organized patterns^8–12^ and may reveal information about the underlying functional circuits^13–18^ or functional connectivity between different brain regions^19–23^. This notion might also be supported by reports that neural activity patterns can vary depending on the context of sensory stimuli, or on motor activity such as eye movement^24–30^.

However, it is technically demanding to distinguish or classify these spatiotemporal activity patterns quantitatively, due to the intrinsic complexity and nonlinearity of neural activities. In most cases, activity patterns in local cortical areas show indefinite or complicated geometric structure that also varies with time dynamically^14,15,31^. For example, propagating waves—one of the most frequently observed patterns—often contain highly nonlinear motion such as compression, reflection^24^, and interactions with other propagating waves^32–36^.

Various approaches have been suggested to address this issue^15,25,37–42^, but an ultimate solution to the problem has not yet been achieved. For example, one idea was that tracing the center of activity mass^15,25^ could successfully provide the trajectory of mean activity, but this approach could not well distinguish spatial features such as convergent or divergent patterns. Another method could analyze the spatial structure of activities by considering the maximal amplitude values at each location^40,43^, but could not handle the temporal change of the amplitude or the dynamic movement during activity. Other studies exploited instantaneous change of activity at each location, using an activity phase latency map, especially for detection of propagating wave-type patterns^38,39^. Even so, this method turned out to be useful only for simple propagating waves without any reflection or spatial oscillation. Last, in a recent study, a mathematical approach based on phase dynamics^41^ was introduced that could classify neural patterns into several stereotypical groups such as sinks or spirals. However, this method showed weak performance for patterns that did not fit into any of the typical groups. Therefore, a more complete and robust method of quantitative classification and labeling of complex neural activity patterns was needed.

**Figure 1:**
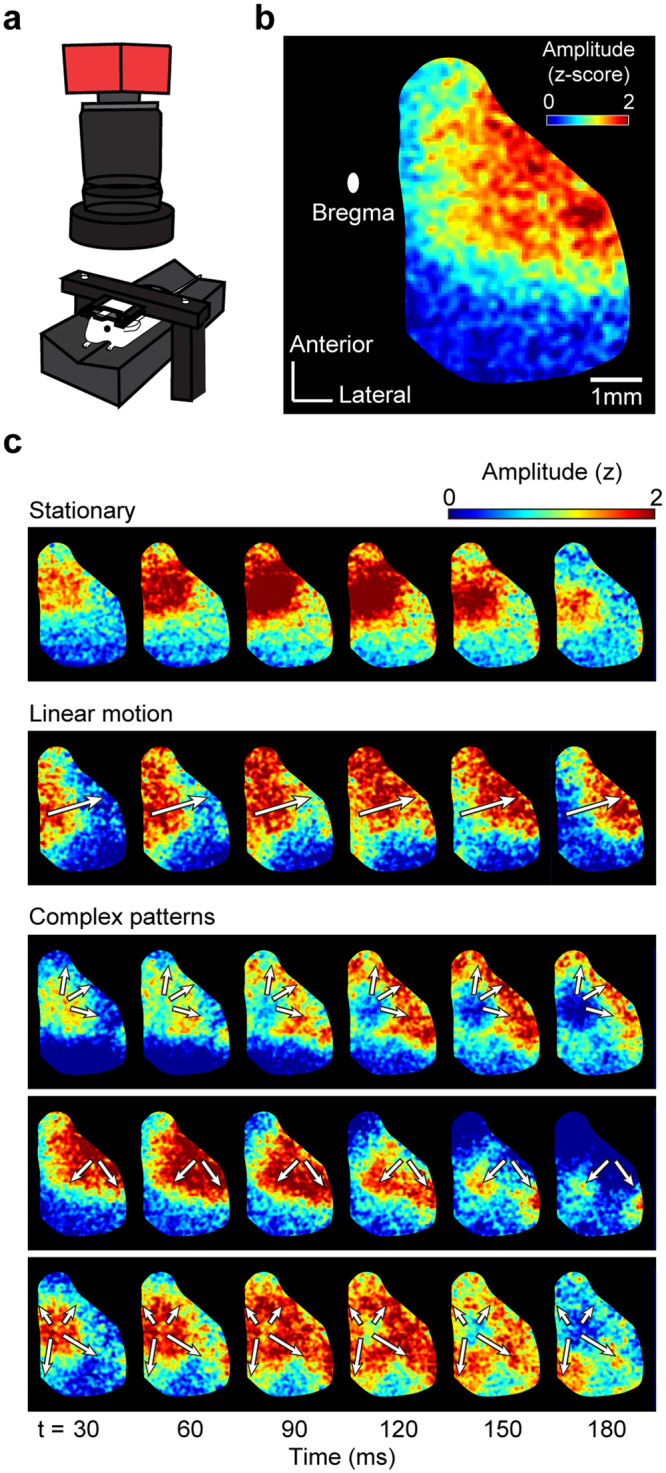
Various spatiotemporal patterns observed in voltage-sensitive dye imaging data. (**a**) Experimental setup for voltage-sensitive dye imaging (VSDI). (**b**) Spontaneous activity of the right hemisphere was measured from each mouse in resting state. Each activity image was aligned in a reference space to match the location of the bregma. The net brain region of interest was set by applying a mask pattern to all imaging data. (**c**) Various spatiotemporal patterns observed from VSDI recordings. Complex patterns including dividing and dispersing motions are more frequently observed than simple patterns such as stationary or linear motion. White arrows illustrate the moving direction of activity.

In this work, we developed a novel method for precise classification and discrimination of spatiotemporal neural activity patterns. Our approach was to categorize neural activity patterns using two independent profiles—the geometric and dynamic profiles—that extract spatial and temporal features, respectively, of given patterns. We first tested our method for classification of computer-simulated neural activities of various types of pattern. We confirmed that our method could successfully distinguish complex forms of spatiotemporal activity patterns composed of complicated spatial and temporal changes, such as convergence and divergence. Then we tested the performance of our method for classification of voltage-sensitive dye imaging (VSDI)^44^ data for Alzheimer’s disease (AD) model and wild type (WT) mice (Fig. 1a,b). In this data set, the features of the activity patterns were hardly observed (Fig. 1c, stationary and linear motion), and in most cases, the patterns observed were highly complex, exhibiting divergence, separation, or merging of patterns (Fig. 1c). However, even under this condition, where differences between the patterns could hardly be detected visually, our method could successfully extract important features of the activity patterns in AD and WT samples, and was able to clearly distinguish all the patterns into two groups.

Our approach provides a solution for precise classification of complex spatiotemporal neural activity patterns. Furthermore, this method is applicable to the analysis of data from various types of optical imaging techniques for a population-level neural activity, regardless of the data collection conditions such as image resolution.

## Results

### Spatiotemporal representation of activity patterns: Geometric and dynamic profiles

**Figure 2:**
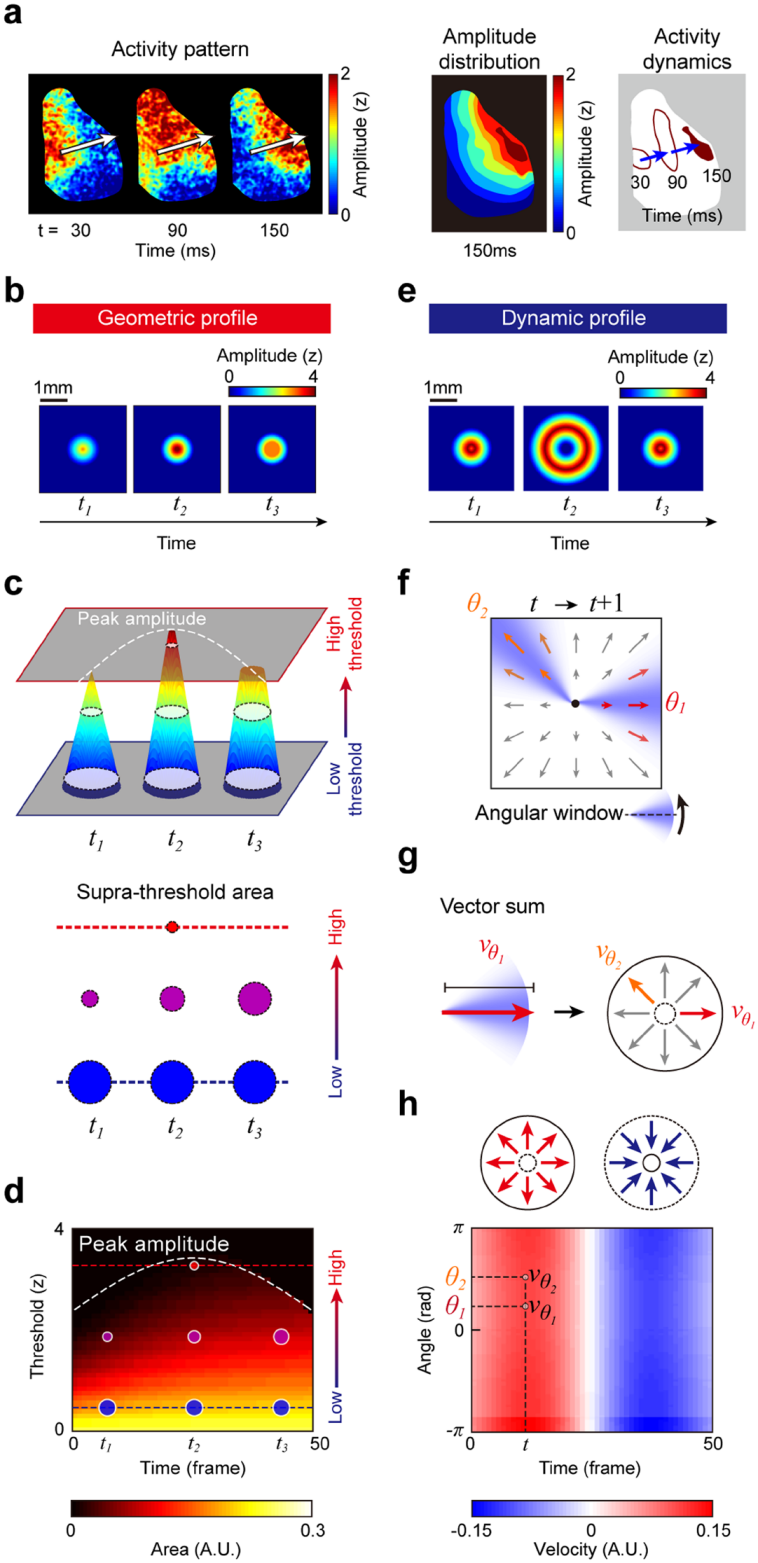
Design of geometric and dynamic profiles. (**a**) Two major components of brain activity patterns: (1) amplitude distribution which represents overall strength and effective size of the activity and (2) the activity dynamics which indicates the spatial changes of the amplitude distribution over time. (**b**–**d**) Design of the geometric profile index. (**b**) A computer-generated sample activity in which the amplitude contour varies but the total area is consistent. (**c**) The supra-threshold area is measured with varying threshold at each time point. At three sample points, t_1_ − t_3_, the supra-threshold areas of the activity appear similar at a low threshold, but appear different as the threshold increases. (**d**) The geometric profile is defined as a 2-D plot of the supra-threshold area with varying threshold at each time point. Colored dots correspond to the supra-threshold area shown in **c**. The white dash line shows the peak amplitude at each moment. (**e**–**h**) Design of the dynamic profile index. (**e**) A computer-generated sample activity propagating outward and inward in the radial direction over time. (**f**) A velocity field extracted from two consecutive frames of image. (**g**) The directional velocity, *v*_*θ*_, is estimated from the weighted sum of velocity field in a Gaussian angular window. The window was rotated around the center of mass of the activity at each time t. (**h**) The dynamic profile is defined as a 2-D plot of the directional velocity for every angle at each time point. The directional velocity is normalized as a ratio to the length of the longer side of the image.

For a precise classification of various spatiotemporal patterns, we considered two major characteristics of neural activities: the topography of an activity amplitude map and its temporal evolution (Fig. 2a). From these geometric and dynamic profiles, we defined our spatiotemporal activity profile index (GeoDyn) to describe quantitatively each type of neural activity pattern.

The geometric profile represents the topographic distribution of activity amplitude at each time. In the previous approaches, the size of neural activity was defined as the area exhibiting more than a certain fixed threshold value of amplitude^45,46^. However, the property of supra-threshold area is highly dependent on the choice of threshold value. For example, for a sample activity in Fig. 2b, the area can appear consistent over time with a low threshold (Fig. 2c). However, the area noticeably varies over time with a high amplitude threshold. Thus, to describe fully the entire profile of an amplitude map, we defined the geometric profile as supra-threshold area with varying threshold (y-axis) at each time point (x-axis) (Fig. 2d). The measured area was normalized using the total area in a recorded frame for scale-free and resolution-independent representation. Such a design enables our geometric profile to contain fully the spatial information of the given patterns.

Next, to classify the dynamic features of activity patterns such as linear motion, radial convergence and divergence over time, we designed a multi-directional profile index that takes into account not only average motion but also the entire dispersion of activity (Fig. 2e–h). For example, when a sample activity pattern propagates outward and inward in the radial direction over time (Fig. 2e), our dynamic profile first calculates a velocity field between two consecutive recording frames (*t*, *t* + 1) by the optic flow method^47^ (Fig. 2f). When the activity propagates outward in the radial direction, all the vectors in a velocity field are pointing outward (positive), while they point inward (negative) during inward propagation. Then, the average velocity in each direction, *v*_*θ*_, was estimated from the weighted vector sum of the velocity field in an angular window where underlying vectors were weighted by the Gaussian function of angle difference from the direction *θ* (Fig. 2g). Then we varied *θ* from −*π* to *π* radians around the center of mass of the activity to construct a two-dimensional (2-D) profile of *v*_*θ*_ computed at each time point (Fig. 2h). In the current example, the index values are positive during outward propagation, while they are negative during inward propagation. For scale-free and resolution-independent analysis of arbitrary images, we normalized the directional velocity as a ratio of spatial distance to the size of the image (i.e., the length of the longer side).

### GeoDyn distinguishes spatiotemporal patterns of noisy artificial neural activities

**Figure 3:**
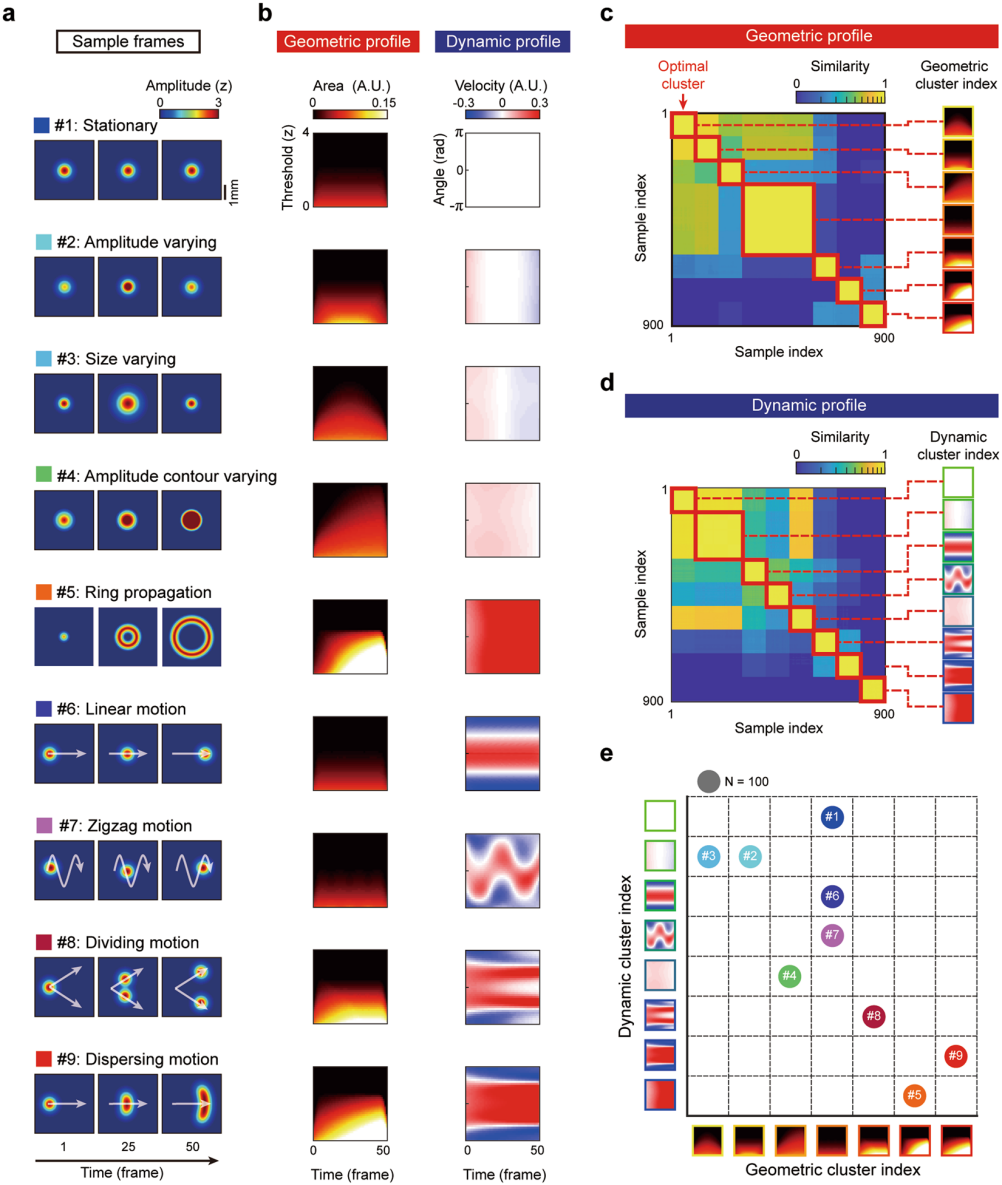
Discrimination of various spatiotemporal activity patterns using the geometric and dynamic profiles. Nine sample activities of various spatiotemporal patterns were analyzed using the geometric and dynamic profiles. (**a**) Each sample was designed to have different patterns of spatiotemporal activity. (**b**) The geometric and dynamic profiles appear different between the samples with distinct features. Nine hundred noisy sample patterns were generated based on nine patterns in **a** (100 samples for each pattern). The geometric and dynamic profiles of selected sample patterns are shown. (**c**) The similarity matrix of geometric profiles for all samples: The sample indices were sorted by the amount of similarity between the pairs. Using optimal hierarchical clustering method, 900 samples were grouped into seven clusters (see Methods). The average of geometric profiles in each group is shown. (**d**) The similarity matrix of dynamic profiles for all samples: The sample indices were sorted as in **c**. By optimal hierarchical clustering, the samples were grouped into eight clusters (see Methods). The average profiles of each group were shown. **(e)** Optimal clustering result of 900 simulated activities. The sample activities were clustered successfully into nine groups.

We tested whether our activity index (GeoDyn), was able to differentiate distinct geometric or dynamic features of computer-generated sample activity patterns, and was able to classify the patterns without any supervised algorithm or pre-set analysis parameters. To mimic various types of observed neural activity in experiments, we generated nine activity patterns (Fig. 3a, see Methods for details) from the combinations of various geometric features: amplitude, size, and amplitude contour change (Fig. 3a, #1–4), and dynamic features: ring-shaped propagation, linear, zigzag, dividing, and dispersing motions (Fig. 3a, #5–9). Then we examined how GeoDyn described these patterns differently.

We observed that geometric and dynamic profiles of each sample pattern were clearly distinguished by their spatiotemporal features (Fig. 3b). Our geometric profile could capture slight differences in activity contour between samples, and the dynamic profile could describe various types of difference between sample activity patterns. For example, the dynamic profile of linear motion activity (Fig. 3a, #6) was clearly distinguished from that of a zigzag motion (Fig. 3a, #7). In our 2-D dynamic profile index, the maxima on the y-axis (angle) at each time step represents the direction of the dominant motion or maximum directional velocity. The trajectory of such a maximum point represents the dominant characteristics of a given motion. For example, a straight-line describes a linear motion and a zigzag shape indicates that the direction of motion switches periodically. Furthermore, bifurcation of the line in sample #8 shows that the activity pattern is divided into two parts, and that the width of the maxima points on the y-axis represents the degree of dispersion of the activity (as shown in #6 and #9). Therefore, these geometric and dynamic profiles contain complete information about the given spatiotemporal patterns in each sample.

Considering these two profiles together allows us to discriminate better the activity patterns of a similar structure. For example, the sample patterns #1, #6, and #7 have exactly the same geometric profiles (Fig. 3a, #1, #6, and #7) because their supra-threshold activities are the same. However, their dynamic profiles are noticeably different in the profile pattern; thus readily separable. Even when the shapes of both geometric and dynamic profiles are similar, GeoDyn can distinguish two patterns from the difference between the amplitudes of the profiles.

Next, to confirm that the GeoDyn was applicable to experimental imaging in which the data on the observed activities was noisy, we tested to see if the method also worked for samples with significant amounts of noise added. For this, we added a Gaussian noise to the nine patterns in Fig. 3a (see Methods for details). Nine hundred sample activities were generated by adding independently generated background noises to the nine template patterns. Then, to determine if these samples of various profiles could be classified into nine groups of source patterns without any supervised algorithm or pre-set parameters, we tried a clustering of activity patterns of similar geometric and dynamic profiles by estimating similarity between the GeoDyn profiles of each sample (Fig. 3c,d, see Methods for details). The optimal hierarchical clustering method^48^ (see Methods and Supplementary Fig. S1) was applied for the classification of each sample. As a result, by simply comparing the geometric and dynamic profiles, we were able to classify successfully 900 noisy activity patterns into nine groups of distinct spatiotemporal characteristics (Fig. 3e). It is noteworthy that the number of clusters (N = 9) in the final result was achieved from simple clustering analysis, not given as an analysis parameter. This shows that our GeoDyn method did well at extracting the underlying principal components of the spatiotemporal patterns in a given activity data set.

In addition, to test the performance of our method for the classification of slightly different activities under noisy conditions, we generated very noisy activity patterns that were hardly distinguishable by visual inspection (Supplementary Fig. S2, Supplementary Video 1–6). In this case, the differences of spatial/temporal parameters such as size, amplitude, and speed between the patterns were set smaller than the level of background noise (i.e., the sigma of the Gaussian). From the classification test of six patterns, we found that our GeoDyn method could successfully distinguish these slight differences in both geometric and dynamic patterns under very noisy conditions (Supplementary Fig. S2). Moreover, each profile clearly showed in which parameter the two patterns were different. For example, differences between the profiles of the Geo index in the bottom area, suggest that two patterns differed in the size of activity. This result shows that our GeoDyn method could successfully distinguish the activities of various samples by precisely comparing the features of their spatiotemporal patterns (Supplementary Fig. S2).

### Discrimination of activity patterns from VSDI recordings of Alzheimer’s disease and wild type mice

**Figure 4:**
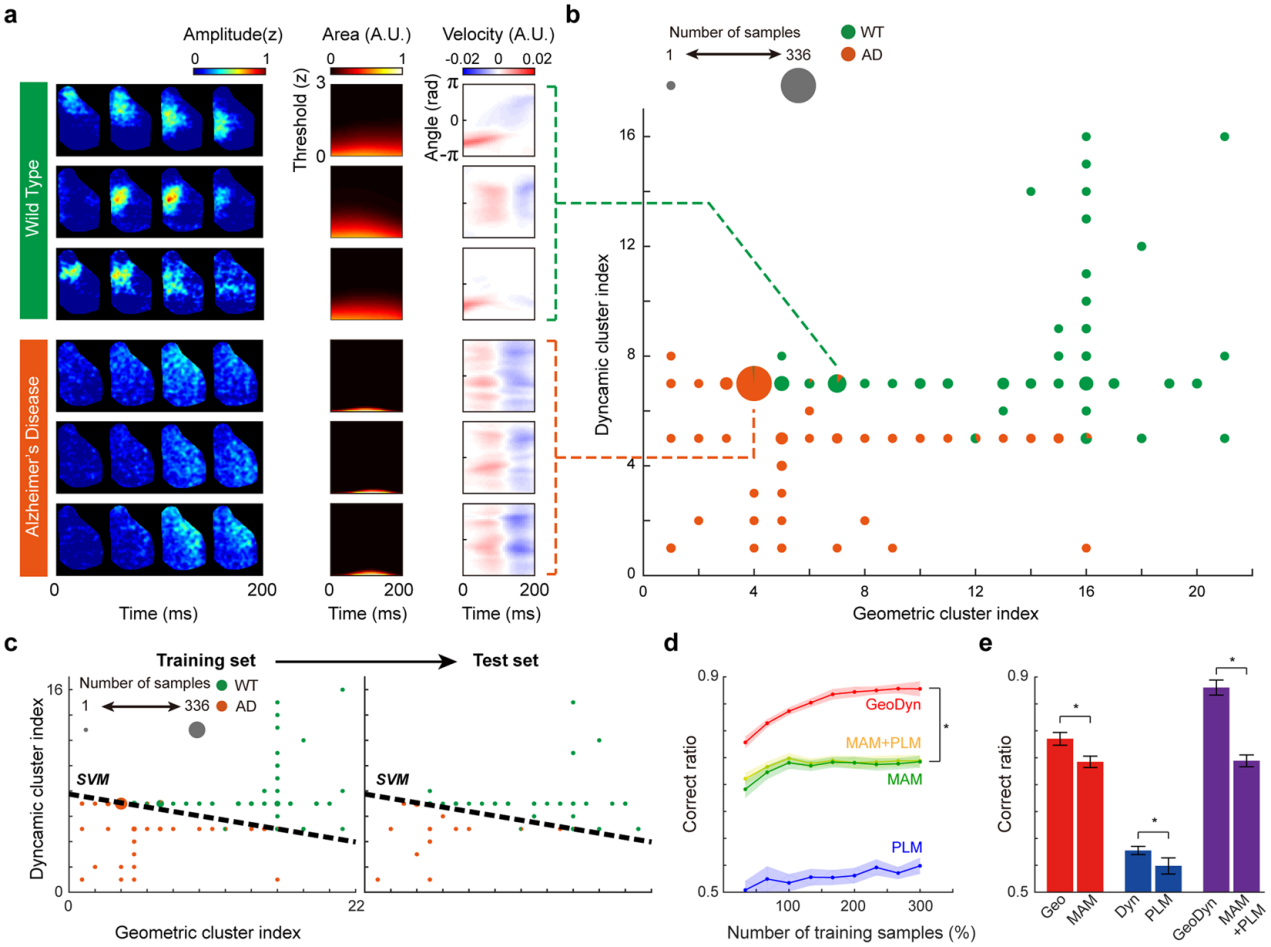
Classification of VSDI data from Alzheimer’s disease (AD) and wild type (WT) mice. (**a**) Sample activity patterns and their GeoDyn profile in AD and WT mice. The activity patterns from the WT mice show much higher amplitude and velocity than for the AD model mice. (**b**) The clustering result of VSDI samples: a total 1200 activity samples are clustered into 67 groups of spatiotemporal patterns. The GeoDyn profiles of selected samples from each type are shown. (**c**) The SVM linear classifier (black dash lines) was trained by the clustering result of a training set. The number of training samples varied from 80 to 720, while the number of test samples was fixed at 240. The order of the geometric and dynamic clusters was sorted using the ratio of a number of AD samples to a number of total samples in each cluster. (**d**) Classification result of activity samples in a test set. Classification performance (mean ± standard error) of four different methods: the GeoDyn profiles (red), the maximum amplitude map (MAM, green)^40^, phase latency map (PLM, blue)^39^ and the combination of MAM and PLM (yellow). The classification test was repeated 100 times for each sampling set (Leave-One-Out cross-validation). Note that the GeoDyn method shows significantly higher performance than the others (*p < 4.883 × 10^−4^, Wilcoxon signed-rank test), regardless of the number of samples in training set. (**e**) Comparison of the classification performance of each method. Note that the geometric profile shows higher correct ratio compared to the MAM (*p < 0.004, Wilcoxon signed-rank test), and the dynamic profile shows higher correct ratio compared to the PLM (*p < 0.011, Wilcoxon signed-rank test). The GeoDyn profiles showed higher correct ratios than did the combination of MAM and PLM (*p < 4.883 × 10^−4^, Wilcoxon signed-rank test) as shown in **d**.

Next, we tested to see if our GeoDyn method could distinguish Alzheimer’s disease (AD) and wild type (WT) mice, using only the difference in the activity patterns from real imaging data. For each type of mouse, spontaneous activity of the right hemisphere was recorded using voltage-sensitive dye imaging (VSDI). For this, 769 samples of activity data were collected from AD mice and 622 samples from WT mice (see Methods for details). To compare the same number of samples between the two types, we randomly selected 600 activity samples for each mouse type. First, we found that the most frequently observed patterns in AD and WT mice appear to have different spatiotemporal characteristics (Fig. 4a). Moreover, the geometric and dynamic profiles of AD and WT activity showed noticeable differences. The geometric profile of WT type activities appeared to have higher amplitude and wider shape than did that of AD type activities. In addition, the WT dynamic profile exhibited higher average velocities than did that of AD mice. Applying the same clustering method for estimated GeoDyn profiles as in Fig. 3c–e, these 1200 activity samples were first classified into 67 pattern groups of distinctive spatiotemporal characteristics (Fig. 4b and Supplementary Fig. S3).

Then, to classify these 67 patterns as belonging to either AD or WT group (Fig. 4b), we used a support vector machine (SVM) linear classifier with the “Leave-One-Out” cross validation algorithm. Specifically, data from a single mouse were chosen as a test set and data from all the other mice were used as training sets, which was repeated for all possible combinations of test and training sets. In this way, the 1200 sample activities were divided into training and test sets. To test the discrimination power of our method, we varied the number of training sets from 80 to 720 samples, while the number of test sets was fixed (n = 240). Then, the SVM linear classifier was trained with the training set for optimal classification of AD and WT type samples. Then, using the trained SVM classifier parameters, the test set samples were classified as AD or WT from their geometric and dynamic profiles (Fig. 4c).

We estimated the average performance of the SVM classifier by repeating this classification test with different training and test sets (N = 100). In addition, we compared performance of our method with two other methods previously suggested: the maximum amplitude map (MAM)^40^ and phase latency map (PLM)^39^ methods. The MAM captures the geometric features of activity patterns by plotting the maximal amplitude of each pixel over time, and the PLM represents the dynamic features by plotting phase latency of each pixel (Supplementary Fig. S6). As a result, the average correct ratio of our GeoDyn using 720 training sets was estimated to be 88.04 ± 1.4% (mean ± standard error). This is significantly higher than that of MAM and PLM regardless of the number of training sets used, and even higher than that of the combined MAM + PLM method to capture both geometric and dynamic features (Fig. 4d, *p < 4.883 × 10^−4^, Wilcoxon signed-rank test). In addition, we also examined the classification performance of each method when we used only a single profile of the activities: the geometric profile, dynamic profile, MAM, or PLM. Even when the geometric and dynamic profiles were used separately, they showed higher performance than MAM and PLM, respectively, in the classification of data by geometric and temporal features. This result explains how our GeoDyn method could perform better than the MAM and PLM; and the combined MAM + PLM, in the classification of WT and AD activities (Fig. 4e, Geo vs. MAM: *p < 0.004, Dyn vs. PLM: *p < 0.011, Wilcoxon signed-rank test).

In the current analysis, we used data from WT and AD mice of comparable ages (N = 7, 4 WT and 3 AD, from 22 to 26 months); however, the activity pattern might vary across individual animals as well as by mouse types. To determine if there is a significant contribution from individual variations, we also performed analysis across individual animals within the same group (Supplementary Fig. S4). From the discrimination test between two mice within each group, our results showed that two mice within the same group were also distinguishable using the GeoDyn indices (both WT vs. WT and AD vs. AD), but its discrimination performance was significantly lower than that between the WT and AD mice (Supplementary Fig. S4a, *p < 8.882 × 10^−16^, Mann-Whitney U-test). Thus, these results suggest that our method can distinguish activity patterns at the level of differentiation of individual animals. In the current data set, however, activity patterns were more readily distinguishable by mouse types than by individual variation.

## Discussion

For this report, we developed a novel method for the classification of various neural activity patterns observed in dynamic imaging data. Our main idea is that both the geometric structure of activity at each time and the dynamic changes of it should be considered together to achieve better classification results. For this, our GeoDyn method defines the geometric and dynamic profiles of each sample, so that different imaging samples can be compared and segregated based on the similarity of their spatiotemporal patterns. In the first test of the method using computer-generated spatiotemporal patterns (Fig. 3), we confirmed that activity patterns of different spatial and/or temporal dynamics appeared noticeably different in our GeoDyn profiles, and thus were readily separable. This result shows that our GeoDyn method could successfully represent distinctive components of spatiotemporal activity patterns.

Next, in the second test using VSDI data from AD and WT mice, we showed how our GeoDyn method could be applied to the classification of real data. When the GeoDyn was estimated from 1200 imaging samples from the AD and WT data, the observed profiles were classified into 67 groups of distinctive spatiotemporal patterns. This means that neural activity in the resting state can vary extremely, even within the same type of AD or WT mice, which makes it hard to distinguish AD and WT samples from their activity pattern analysis. However, using our GeoDyn method, similar spatial (temporal) patterns have similar geometric (dynamic) profiles, so that the 67 profile patterns could be re-grouped as AD or WT according to their similarity (Fig. 4b).

From the inspection of profile indices from AD and WT samples (Fig. 4a,b), we found that the geometric profiles contrasted more between AD and WT samples than their dynamic profiles did. This suggests that, in this set of data, geometric features of the activity patterns might be more informative for the classification of samples. This was also supported by the classification results from other methods (MAM and PLM) that used different components of geometric and dynamic information (Fig. 4d,e and Supplementary Fig. S5). The MAM method mostly measures geometric distribution of patterns, and showed higher classification performance than did the PLM method, which measures the dynamic propagation of patterns. On the other hand, our GeoDyn method considers both geometric and dynamic profile information and showed a significantly higher correct ratio than did the other two methods.

To see whether applying both the MAM and PLM could enhance performance, we performed the classification using the combined MAM + PLM method. The performance of the combined method was still lower than that of the GeoDyn and similar to that of the MAM only. This means that the PLM could not contribute much to the classification of WT and AD (Supplementary Fig. S9). This is also consistent with the result that the geometric profiles differed more between AD and WT samples than did their dynamic profiles. This result suggests that, first, the GeoDyn method can make better use of spatiotemporal activity information than the previously suggested methods. Second, although AD and WT mice seem to generate various types of spatiotemporal profiles, the activity patterns within each group share some geometric and dynamic features, which can be used to distinguish the activity patterns across the two groups.

We suggest that our GeoDyn can contribute significantly to the study on the relationship between the variation of neural activity and the changes in underlying neural circuitry. Previously, there have been a number of studies observing various patterns of neural activities in the brain, and emphasizing their importance in studying neural networks and the connectivity of neural circuitries^13,14,22,49^. In most cases, the observed activity patterns were considered to reflect some important biological meanings, such as information about local neural circuit or long-range network interaction between different brain regions. For example, one suggested that the activity propagation measured from VSDI was highly correlated with axonal projection in the mouse brain^15^. From the current study, we propose that such biological meaning of neural activity patterns might be effectively examined using GeoDyn profile analysis. As an example, we might ask a sample question such as “Is there any sign of structural difference in the circuitry of WT and AD type mouse brain?” From the profiles of the average sample-activity of the WT and AD mice (Supplementary Fig. S5a,b), it was clear that the differences between the two dynamic profiles of WT and AD were not significant in their shape. However, their amplitudes were noticeably different (Supplementary Fig. S5c,d). Our analysis also indicated that the activities from the two types of mice differed from their dispersion, but that their propagation speed appeared to be similar. If the propagation patterns were correlated with neural connectivity in the network, we could speculate that the functional connectivity might be modulated in AD mice by some factor. In addition, the difference between the geometric profiles could imply that their neural firing rate or excitability might be different locally. Although the GeoDyn method could help to examine candidates for the biological basis of activity variation, how to relate arbitrary patterns of spatiotemporal features to a direct biological basis remains elusive. Further study of well-designed activity observations with anatomical circuitry analysis is necessary to validate the assumption of a biological basis for the different patterns in WT and AD data. The current study was focused on the development of an analysis algorithm, so we leave this issue for future study.

Our result also shows that the GeoDyn method can successfully classify neural activity patterns even in the resting state, without any control of sensory input stimuli. In previous studies of VSDI data^39^, neural activities were measured under highly specific conditions of visual stimuli. In such conditions, neural activities show very limited variation in their spatiotemporal profiles. This may be helpful for simplicity in neural activity pattern analysis, but cannot fully explain the various features of neural activity observed under conditions that are more realistic. Here, we showed that our GeoDyn method could readily classify various activity patterns achieved with no input control. Thus, our analysis method appears to be applicable to the classification of any imaging data from realistic conditions. In general, imaging data from different conditions or methods have different spatiotemporal resolution or regions of interest (ROI). This issue can readily be addressed by our GeoDyn method because it extracts a common feature from spatiotemporal patterns regardless of the size or resolution of the imaging data. This enables us to apply our method to imaging data at different spatial and temporal scales. Therefore, our method might be a strong tool for the comparison of neural imaging data from different conditions, different brain regions, or even from different species.

Last, classification of neural activity patterns might also be performed using various types of machine learning techniques. Our GeoDyn method can be used not only as an independent pattern classifier, but also as a pre-processor of raw data to be applied in machine-learning analysis. Raw image data from different experimental environments and subject conditions have various scale and dimension parameters (e.g., brain shape and size), but our GeoDyn gains an advantage by converting raw data at various scales into a scale-free normalized profile. Such pre-processing makes it easier to apply the machine learning techniques used to enhance the classification performance. In addition, GeoDyn was designed to provide information about spatiotemporal patterns by parametrizing geometric and dynamic features of activity (such as speed or size). This means it could help with post-analysis of the classification results from machine learning, such as finding pattern motifs or a biological basis. While machine-learning algorithms can also classify various activity patterns well, they require additional analytical processes to extract physical or biological meanings from the results achieved. Our GeoDyn method can make processes involving machine-learning techniques much simpler.

## Methods

### Animals

Four WT (C57BL/6) mice (all 22 months old) and three transgenic AD model (APP_SWE_/PS1_ΔE9_) mice (22, 24 and 26 months old, respectively) were used for the experiments. They were co-housed in air-conditioned cages under a 12:12 hour light:dark cycle. Free access to UV sterilized water and food was given. All animal experiments were performed in accordance with the guidelines and policies for rodent experimentation provided by the KAIST Institutional Animal Care and Use Committee (IACUC). The protocol used was approved by the IACUC of KAIST (IACUC-14-134).

### Surgical Procedure

For anesthesia induction, 3% isoflurane was used and 1–1.5% isoflurane with 100% oxygen was used during surgery. During data collection, 0.5–0.75% isoflurane was used. Head plates were custom designed and fixed to the skull with dental cement (Bosworth Trim II, Keystone Industries, Gibbstown, NJ, U.S.A.) and cyanoacrylate glue (Loctite 401, Henkel, Düsseldorf, Germany). The head plate was then fastened to a metal frame. A large portion of the right hemisphere of the cortex was exposed with a 7 × 6 mm unilateral craniotomy following previous literature. The dura mater was removed with a Vannas micro-scissor (FST, Vancouver, Canada) and fine forceps (FST, Vancouver, Canada). Extreme care was taken to prevent damage to the cortex. A heating pad and a feedback rectal probe (TCAT-2LV, Physitemp instruments, Clifton, NJ, USA) were used to maintain the body temperature at 37 °C throughout the experiment.

### Voltage-Sensitive Dye Imaging (VSDI)

Artificial cerebrospinal fluid (aCSF) of pH 7.4 and temperature of 37 °C was used to prepare RH1692 dye (Optical Imaging, Rehovot, Israel) solution (1 mg/ml). Bath application of the dye to the exposed cortex was performed for 60–90 min. The unbound dye was washed away by loading the cortex with aCSF for 30 min after the bath application. Then, 1.5–2% agarose was used to cover the cortex to minimize pulsation and movement artifacts. A 12 mm coverslip was put onto the agarose layer before agarose cooling. The coverslip was then fixed to the head plate with cyanoacrylate glue. A 100 W halogen lamp focused at 400 µm from the cortical surface was used for excitation. The excitation light was filtered with a filter centered at 632 nm (FF02-632/22-25, Semrock, NY, USA) and was reflected onto the cortex using a light guide. The signals coming back from the cortex were collected using a tandem lens macroscope with a long-pass emission filter at 675 nm (84–753, Edmund Optics, Barrington, NJ, USA). The macroscope was connected to a CCD (MV1-D1312-160-CL-12, PhotonFocus, Lachen, Switzerland) that recorded the signals at 150 Hz using a CELOX imaging system and VDAQ software (Optical Imaging, Rehovot, Israel). The data had a spatial resolution of 62.5 μm per pixel.

### Pre-processing VSDI images

We collected 370 imaging epochs of 20 s each from the two mice groups (n = 267 from WT and 103 from AD mice, respectively), which were aligned in a 2-D reference space to match the anterior-posterior axis of the brain using a custom built MATLAB function (Fig. 1b). To select a net brain region of interest (ROI) observed in all mice, a mask pattern was applied to achieve the ROI for analysis of all samples. Edge pixels of low signal-to-noise ratio were removed. The amplitudes of activity were z-scored by the mean and standard deviation of the whole activity. To eliminate high-frequency noise signals, each epoch was filtered using a zero-phase band-pass filter at 0.1–6 Hz. To specify significant activity patterns only, the average activity value over time was calculated in each epoch and the intervals of which the amplitude exceeded the threshold (0.5 of z-score) were extracted. In addition, activities that do not show any spatial patterns were excluded.

### Generation of simulated activity pattern

Nine sample activity-patterns were generated from computer simulations using the 2-D Gaussian kernel (Eq. 1) or a truncated cone function (Eq. 2) as a cone with an apex cut off, to mimic the observed activity patterns in the imaging data:

1 $$u(x,y,t)=A(t)\cdot \exp (-\frac{{(x-{x}_{0}(t))}^{2}+{(y-{y}_{0}(t))}^{2}}{2{\sigma }_{A}{(t)}^{2}})$$

2 $$u(x,y,t)=min\{A(t),A(t)\cdot (\frac{{r}_{2}(t)-\sqrt{{(x-{x}_{0}(t))}^{2}+{(y-{y}_{0}(t))}^{2}}}{{r}_{2}(t)-{r}_{1}(t)})\}$$

where *u*(*x*, *y*, *t*) is the activity amplitude at spatial position x and y at time point t. A(t) is the peak amplitude at $[({x}_{0}(t),{y}_{0}(t)],$ ${\sigma }_{A}(t)$ represents the standard deviation of the Gaussian kernel, *r*_1_(*t*) and *r*_*2*_(t) are the top and bottom radii of the truncated cone function. All samples were designed with a size of 101 × 101 pixels and 50 time frames. The equations of each activity pattern (with detailed parameters) are listed in Table 1.

**Table 1:** Equations of each activity pattern.

Pattern number	Pattern types	${\boldsymbol{u}}({\boldsymbol{x}},{\boldsymbol{y}},{\boldsymbol{t}})$
#1	Stationary	$3\cdot exp(-({(x-51)}^{2}+{(y-51)}^{2})/(2\cdot {7.5}^{2}))$
#2	Amplitude varying	$(2+3sin(\pi t/50))\cdot exp(-({(x-51)}^{2}+{(y-51)}^{2})/(2\cdot {7.5}^{2}))$
#3	Size varying	$3\cdot exp(-({(x-51)}^{2}+{(y-51)}^{2})/(2\cdot {(7.5+7.5sin(\pi t/50))}^{2}))$
#4	Amplitude contour varying	$min\{4,(4+0.04t)\cdot ((0.22t-\sqrt{{x}^{2}+{y}^{2}})/(0.22t-15))\}$
#5	Ring propagation	$max\{0,\,4-0.2\cdot abs(\sqrt{{x}^{2}+{y}^{2}}-(0.6t))\}$
#6	Linear motion	$3\cdot exp(-({(x-(21+1.2t))}^{2}+{(y-51)}^{2})/(2\cdot {7.5}^{2}))$
#7	Zigzag motion	$3\cdot exp(-({(x-(21+1.2t))}^{2}+{(y-(51+12sin(\pi t/25)))}^{2})/(2\cdot {7.5}^{2}))$
#8	Dividing motion	$min(\sum _{\theta }3\cdot exp(-({(x-(21+1.2cos(\theta )t))}^{2}+{(y-(51+1.2sin(\theta )t))}^{2})/(2\cdot {7.5}^{2})),3)\theta =(-\pi /6,\pi /6)$
#9	Dispersing motion	$$\begin{array}{c}min({\sum }_{\theta }3\cdot exp(-({(x-(21+1.2cos(\theta )t))}^{2}+{(y-(51+1.2sin(\theta )t))}^{2})/(2\cdot {7.5}^{2})),3)\\ \theta =(n\pi /120-\pi /6\,|\,n=0,\,1,\,2\,\cdots 19,\,20)\end{array}$$

To design a noisy version of the activity patterns, we added background Gaussian noise to the activity. The mean and standard deviation were set to zero and one, respectively. The amount of noise added was set from visual inspection so that the similar-but-different activity patterns (e.g., Supplementary Fig. S2, #2 and #3; #5 and #6) were visually indistinguishable (Supplementary Videos 1–6).

### Analysis of Geometric and Dynamic Features of Neural Activity

#### Design of the Geometric and Dynamic profiles (GeoDyn)

For a geometric profile, the range of the threshold value for calculating the supra-threshold area of activity was set from ‘0’ to ‘5’ with 0.1 intervals in the z-score. For a dynamic profile, the velocity field was calculated from two consecutive frames using the combined global-local algorithm of the optical flow method^47^. The MATLAB toolbox (Mathworks, USA) for the optical flow method was adapted from an open source online^50^. The directional velocity, *v*_θ_, was calculated as the weighted sum of the velocity field within a given angular window (Fig. 2h). The angular window, f(*ϕ*), was designed with a Gaussian kernel that varied through the polar axis (*ϕ*) (Eq. 3):

3 $${\rm{f}}(\varphi )=\frac{1}{\sqrt{2{\sigma }_{\theta }^{2}\pi }}\exp (-\frac{{(\varphi -\theta )}^{2}}{2{\sigma }_{\theta }^{2}})$$

where *θ* represents the center of the window, which rotates from 0° to 360° with 7.5° intervals. The standard deviation of the Gaussian kernel, *σ*_*θ*_ was set as 7.5°.

#### Similarity measurement by geometric or dynamic profiles between different samples

To estimate similarity between geometric (or dynamic) profiles of different samples, we first calculated the squared error between the profiles. The maximum error value from the combinations of all sample profiles was normalized to ‘1’. Then, similarity was defined as the difference between the normalized squared error and ‘1’ (maximum error). To consider any possibility of temporal phase difference between two activity patterns, we measured the similarity while shifting one profile along the time axis, where the amount of time shifting was limited to less than a quarter of the shorter activity pattern duration. Then, the similarity between the two profiles was finally chosen as the maximum among those values.

#### Hierarchical clustering of pattern groups

A schematic of hierarchical clustering is shown in Supplementary Fig. S1. The dendrogram (Supplementary Fig. S1, right) shows the procedure of hierarchical clustering used to group the samples based on similarity between samples (Supplementary Fig. S1, left). The vertical axis represents the sample objects and the horizontal axis indicates the similarity between the cluster nodes (black circles). The horizontal location of each cluster node indicates the averaged similarity between samples of two leaf cluster nodes. The cluster nodes were merged hierarchically by their similarity: the left ends of cluster nodes represent each single sample, while the right ends represent all the samples in one cluster group. Finally, by varying the similarity cutoff, we could optimize the number of clusters that maximized similarity within each group, but minimized it across the groups. This process was performed using the “linkage” function in MATLAB, where the cutoff value was set to minimize the cluster validation index, i.e. the cluster balance^48^ (Eq. 4).

4 $$Cluster\,balance=Intra\,cluster\,error+Inter\,cluster\,error$$

5 $$Intra\,cluster\,error=\sum _{h=1}^{n}\sum _{i\in {C}_{h}}\sum _{j\in {C}_{h}(j\ne i)}\frac{{d}_{ij}^{2}}{2|{C}_{i}|}$$

6 $$Inter\,cluster\,error=\frac{1}{n}\sum _{h=1}^{n}\sum _{i=h}^{n}\sum _{j\in {C}_{h}}\sum _{k\in {C}_{i}}\frac{{d}_{jk}^{2}}{|{C}_{j}||{C}_{k}|}$$

The above equations show the calculation of cluster balance when all the samples are grouped into n clusters, [*C*_1_, *C*_2_…*C*_*n*_]. Here, $|{C}_{i}|$ is the number of samples in *C*_*i*_, *d*_*ij*_ means 1 – the similarity between the sample i and j. After optimal clustering, the clusters containing a single sample were considered one group for simplicity.

#### Support Vector Machine (SVM) linear classifier

For the training of the support vector machine (SVM), the clustering result of the training set was provided, including the indices of geometric and dynamic clusters of each sample. We used the “fitcsvm” function in MATLAB to train the SVM classifier. Then, the classification of the test-set type was performed using the “predict” function based on the trained SVM classifier. We also applied a standard “Leave-One-Out” cross-validation to perform a SVM classification correctly: An individual mouse was chosen as a test set and all the other mice were used as training sets. We repeated this sampling 100 times for each of 12 cases (12 possible combination pairs from 4 WT and 3AD mice). Before training the SVM classifier using real data, the geometric and dynamic indices were sorted in ascending order according to the value of $(\frac{{\rm{number}}\,{\rm{of}}\,{\rm{AD}}\,{\rm{samples}}\,{\rm{in}}\,{\rm{cluster}}}{{\rm{number}}\,{\rm{of}}\,{\rm{total}}\,{\rm{samples}}\,{\rm{in}}\,{\rm{cluster}}})$, to perform the classification correctly.

## Electronic supplementary material

Supplementary Information

Supplementary Video 1

Supplementary Video 2

Supplementary Video 3

Supplementary Video 4

Supplementary Video 5

Supplementary Video 6
