# Supplementary material for: Classification of Spatiotemporal Neural Activity Patterns in Brain Imaging Data
Source: Sci Rep. 2018 May 29;8:8231. doi: 10.1038/s41598-018-26605-z (PMC5974089; doi:10.1038/s41598-018-26605-z)
Supplement: Supplementary file 1 — Supplementary Information [file 41598_2018_26605_MOESM1_ESM.pdf]

Classification of Spatiotemporal Neural Activity Patterns in Brain Imaging Data

Min Song[1,2†], Minseok Kang[1†], Hyeonsu Lee[1], Yong Jeong[1,2,*] and Se-Bum Paik[1,2*]

[1]Department of Bio and Brain Engineering, [2]Program of Brain and Cognitive Engineering, KAIST, Daejeon 34141, Republic of Korea

†These authors contributed equally on this work.

Correspondence should be addressed to Yong Jeong (yong@kaist.ac.kr) or Se-Bum Paik (sbpaik@kaist.ac.kr)

Supplementary information

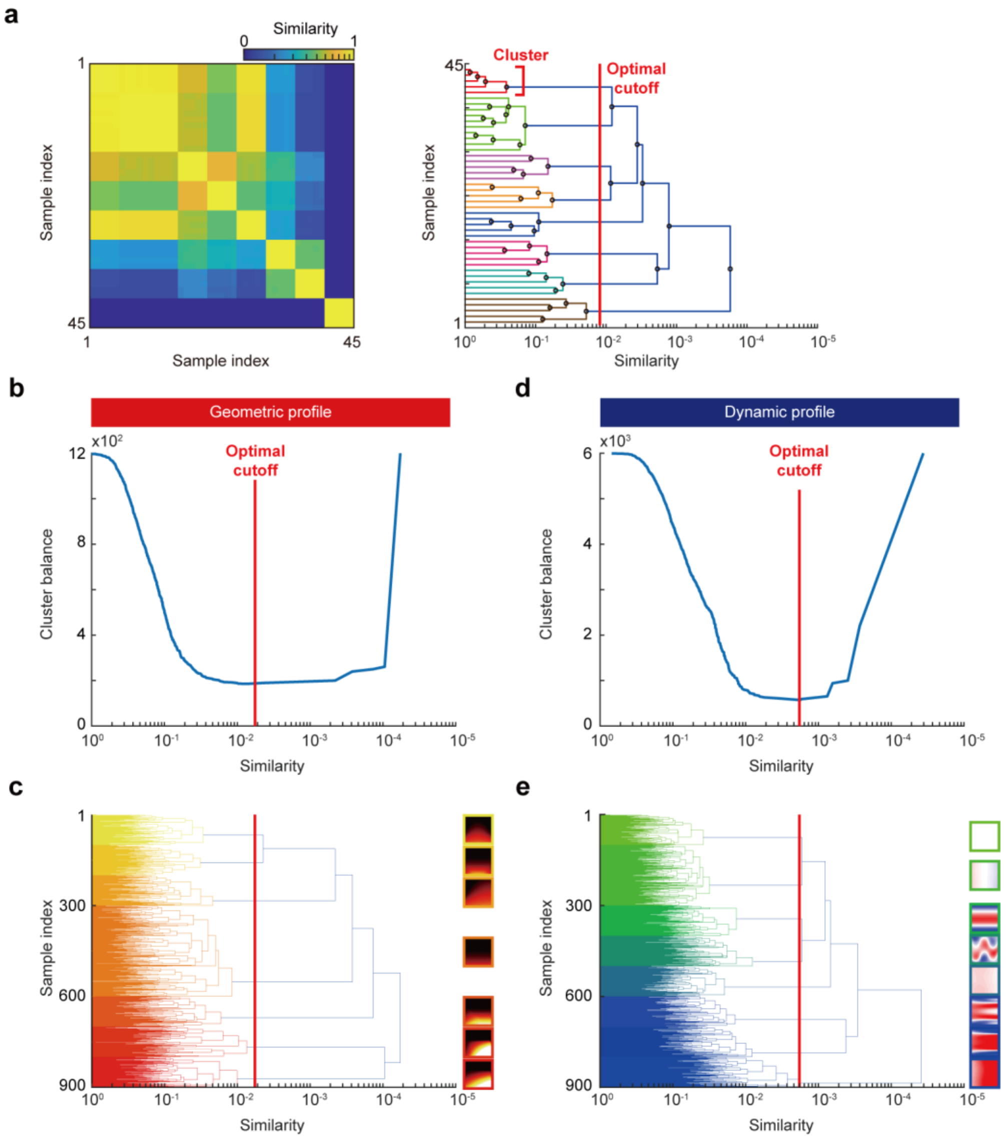

Supplementary Figure S1. Hierarchical clustering of simulated neural activity samples

(a) (Left) Similarity matrix of 45 sample activities, (Right) Dendrogram plot of the samples: Sample pairs with similarity larger than the cutoff value are considered in the same group. **(b)** The "cluster balance" (see Methods) for clustering validation was estimated with varying cutoff values to find an optimal clustering of geometric profiles. The optimal cutoff value (red solid line) was set to minimize the cluster balance. **(c)** Clustering result of geometric profiles using an optimal cutoff value. **(d)** The cluster balance was estimated with varying cutoff values to find an optimal clustering of dynamic profiles. **(e)** Clustering result of dynamic profiles using an optimal cutoff value.

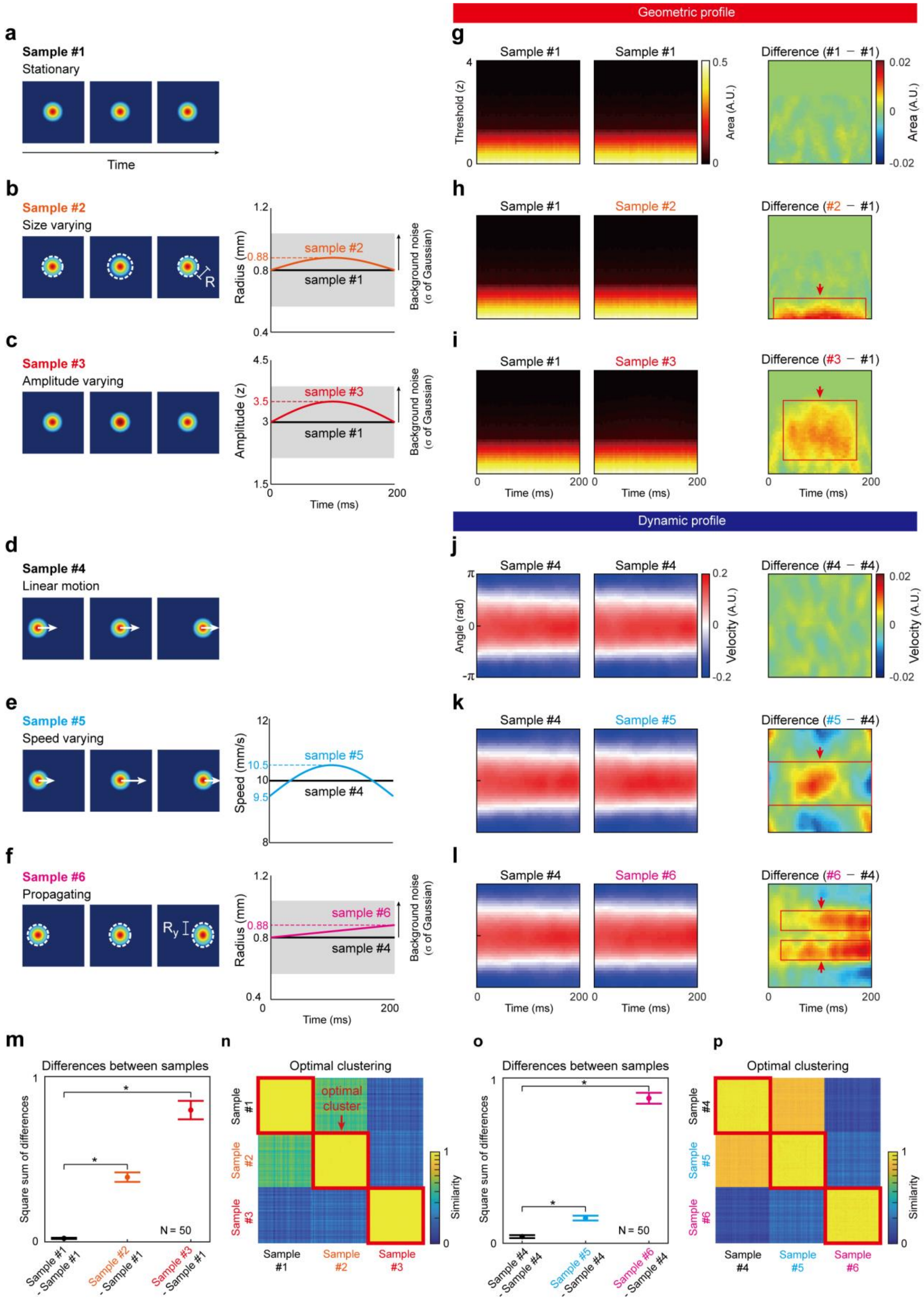
Geometric profile
Dynamic profile
a
Sample #1
Stationary
Time
b
Sample #2
Size varying
R
Radius (mm)
sample #2
sample #1
0.88
0.8
Background noise (σ of Gaussian)
c
Sample #3
Amplitude varying
Amplitude (z)
sample #3
sample #1
3.5
3
Time (ms)
d
Sample #4
Linear motion
e
Sample #5
Speed varying
Speed (mm/s)
sample #5
sample #4
10.5
10
9.5
f
Sample #6
Propagating
R_y
sample #6
sample #4
g
Sample #1
Sample #1
Difference (#1 − #1)
Threshold (z)
Area (A.U.)
h
Sample #2
Difference (#2 − #1)
i
Sample #3
Difference (#3 − #1)
j
Sample #4
Sample #4
Difference (#4 − #4)
Angle (rad)
Velocity (A.U.)
k
Sample #5
Difference (#5 − #4)
l
Sample #6
Difference (#6 − #4)
m
Differences between samples
Square sum of differences
N = 50
Sample #1 - Sample #1
Sample #2 - Sample #1
Sample #3 - Sample #1
n
Optimal clustering
optimal cluster
Similarity
o
Differences between samples
Sample #4 - Sample #4
Sample #5 - Sample #4
Sample #6 - Sample #4
p
Optimal clustering

Supplementary Figure S2. Classification of the sample patterns of various spatial and temporal features

(a–c) Comparison of the sample activities with slight variations of spatial patterns: **(a)** Stationary, **(b)** Size varying from **a**, and **(c)** Peak amplitude varying from **a**. Note that the variation of each parameter is within the background noise level (the standard deviation of Gaussian noise). **(d–f)** Comparison of the sample activities with slight variations in temporal patterns: **(d)** Linear motion, **(e)** Speed varying from **d**, and **(f)** Activity area propagating from **d**. Note that variations of the radius are smaller than the background noise level **(g–i).** The geometric profiles of sample patterns in **a–c**. **(g)** Geometric profiles from the two identical patterns in **a,** estimate the amount of variance in the background noise. **(h)** The difference between Samples #1 and #2 is observed in the bottom area of the geometric index (red arrow), and shows that the two patterns are different in size. **(i)** The difference between Sample #1 and #3 is observed in the high threshold area of the geometric index (red arrow), and shows that the two patterns are different in amplitude. **(j–l)** The dynamic profiles of the sample patterns in **d–f**. **(j)** Dynamic profiles from the two identical patterns in **d,** estimate the amount of variance in the background noise. **(k)** The difference between Sample #4 and #5 is observed in the center area of the dynamic index (red arrow), and shows that the two patterns are different in their speed in the horizontal direction. **(l)** The difference between Sample #4 and #6 is slightly off from the center angle of the dynamic index and increases over time (red arrows). This shows that the two patterns are mostly different in the surrounding area of the activity, away from the center. **(m)** Differences between the geometric profiles of Samples #1–#3. Note that Samples #2 and #3 are clearly distinguishable from Sample #1, even though their variation is within the noise level. **(n)** Optimal clustering of geometric profiles from 150 samples (50 samples for each pattern). **(o)** Differences between the dynamic profiles of Samples #4–#6. **(p)** Optimal clustering of dynamic profiles from 150 samples (50 samples for each pattern).

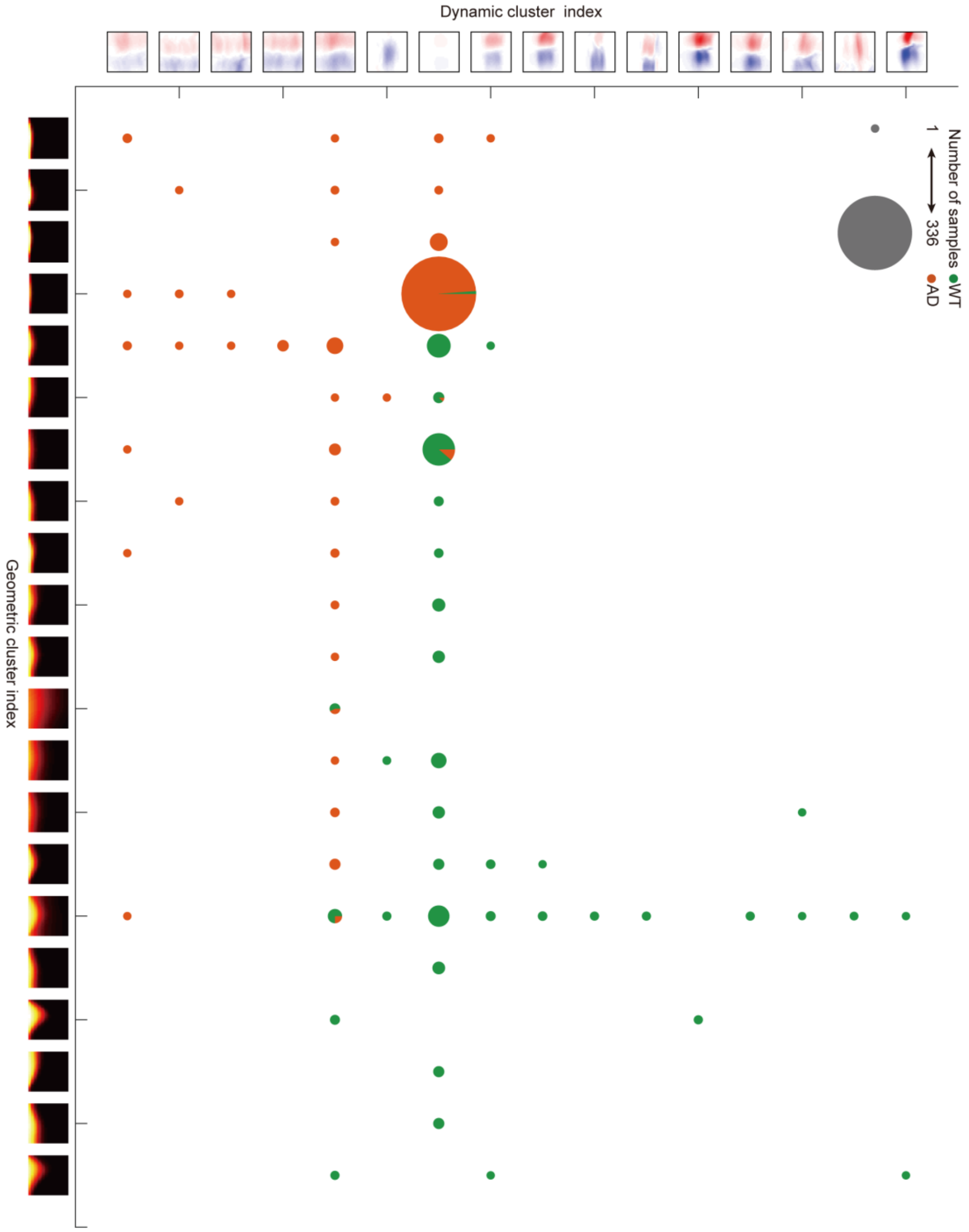

Supplementary Figure S3. Clustering result of VSDI samples

Extended from Figure 4 b, the averaged profiles of each cluster index were displayed along the x and y axes. Each pie graph represents a ratio of the number of AD and WT samples belonging to each geometric and dynamic profile pair, orange and green colors indicate AD and WT, respectively. Size of circles indicates the number of samples grouped in each geometric and dynamic profile pair.

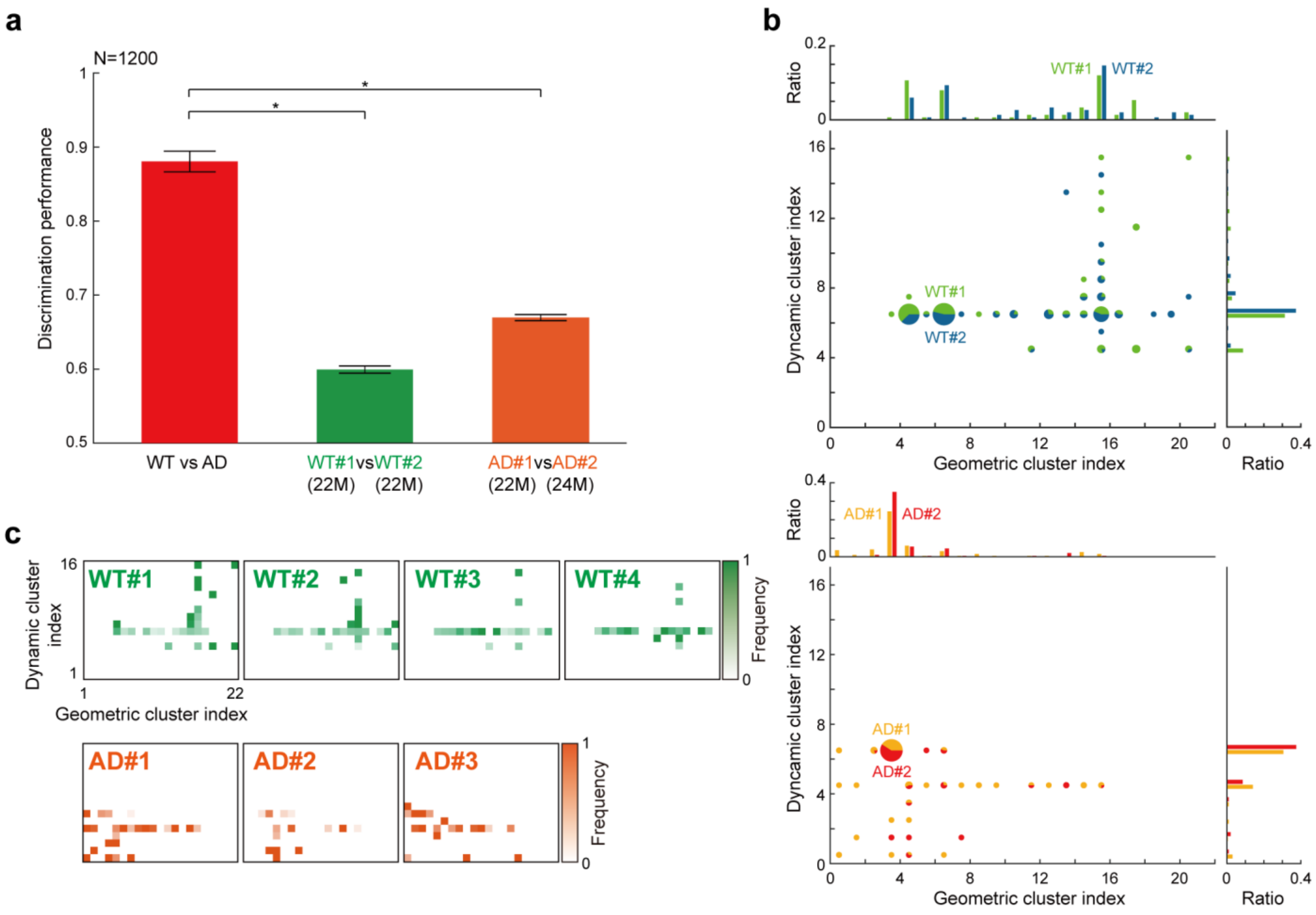

Supplementary Figure S4. Discrimination by mouse type and individual variance

(a) Discrimination test under various conditions. Discrimination performance was defined as the average correct ratio of classification (mean ± standard error), when the number of training sets is triple the number of test sets. Note that the discrimination performance was significantly better for the classification of WT and AD mice groups, compared to the classification of animals within the same group (Mann-Whitney U-test, $*p < 8.882\times10^{-16}$). **(b)** Comparison of the GeoDyn profiles of two WT-mouse samples and two AD-mouse samples. Large overlap of the two distributions indicates that the activity patterns of the two mice are similar. **(c)** GeoDyn profiles of individual WT and AD mice (N = 7).

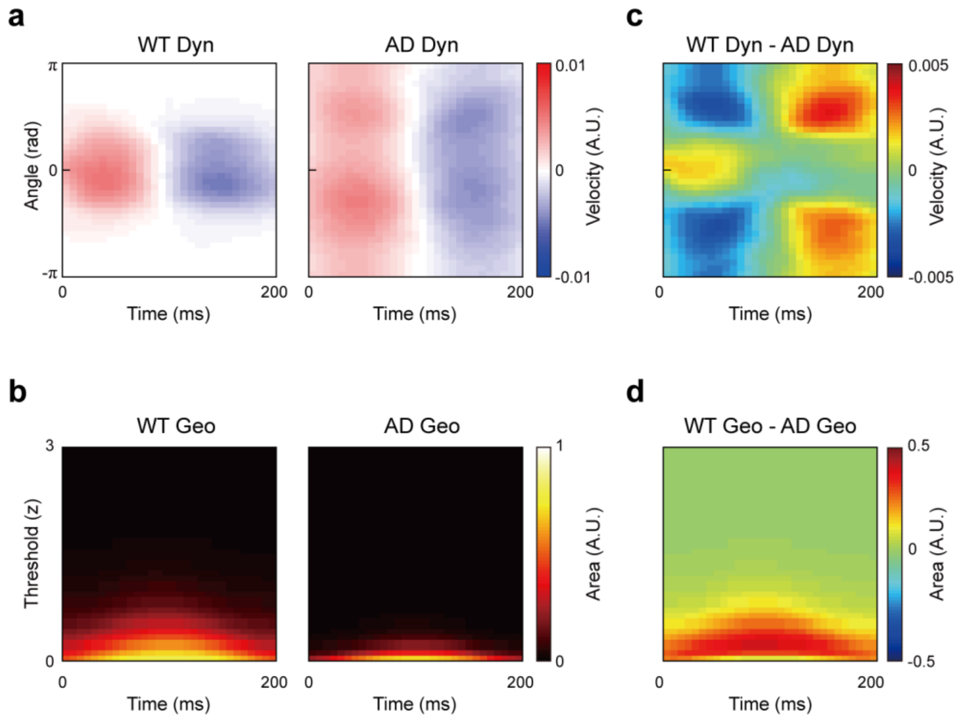

Supplementary Figure S5. GeoDyn profiles of the representative pattern of WT and AD groups

(a) Average dynamic profiles of WT and AD mice. The two profiles are similar in shape, but different in their amplitude. **(b)** Average geometric profiles of WT and AD mice. The two profiles are different in both their area shape and amplitude. **(c)** Differences between the profiles in **a**. **(d)** Differences between the profiles in **b**.

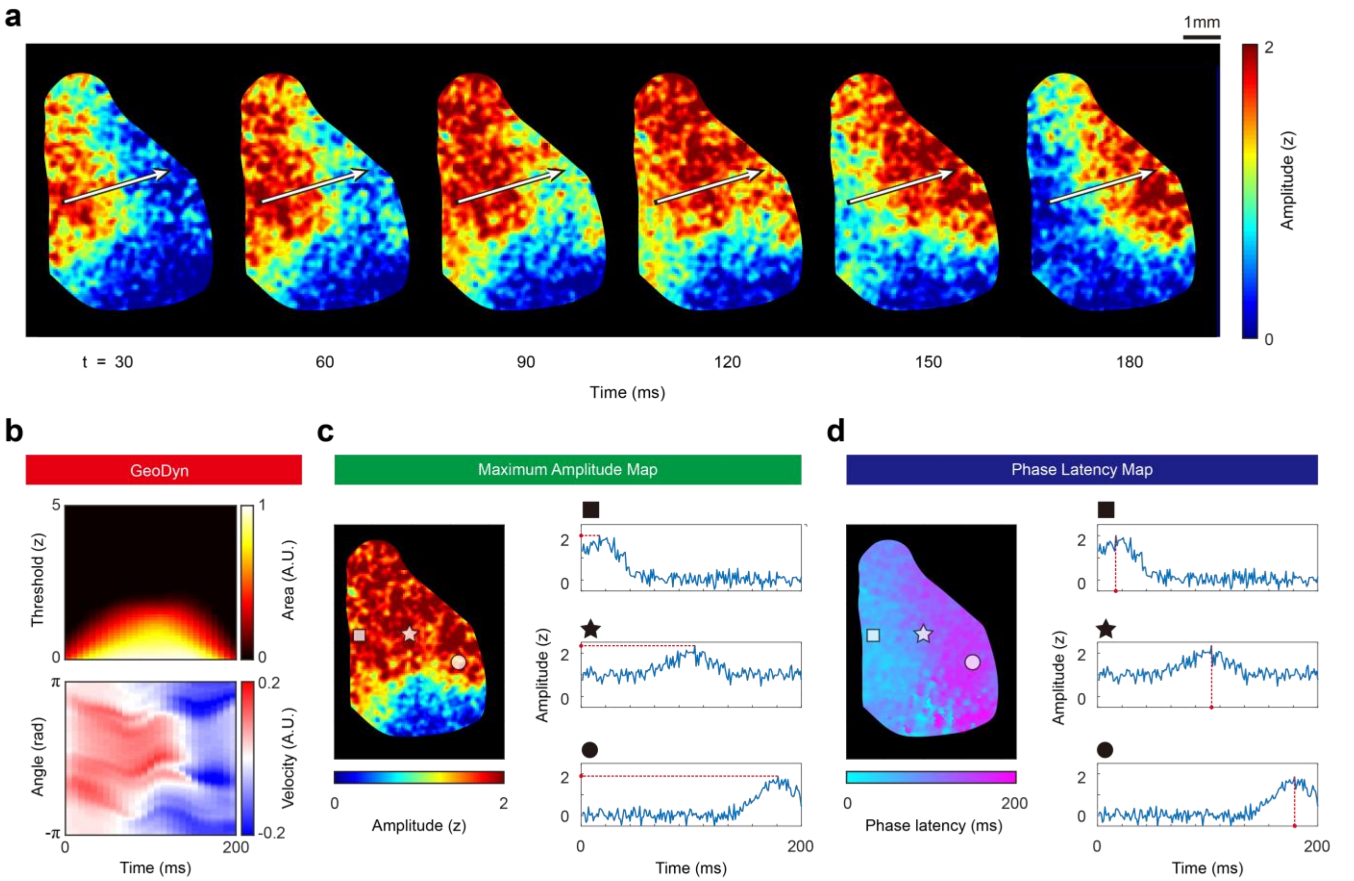

Supplementary Figure S6. Example of GeoDyn, MAM, and PLM analysis

(a) A VSDI sample showing linear motion pattern. **(b)** The geometric and dynamic profiles of the sample activity in **a**. **(c)** The maximum amplitude map (MAM) of the sample activity in **a** (left). Activities at selected locations (■, ★, ●) were plotted (right). MAM displays the maximum amplitude of activity over time at each position. Value of the maximum amplitude at each position (red dash lines) is plotted in the MAM. **(d)** The phase latency map (PLM) of the sample activity: PLM is a plot of phase latency of each position (left). Activities at selected locations (■, ★, ●) were plotted (right). The phase latency at each position (red dash lines) is plotted in the PLM; thus, it represents the overall propagation pattern of activity.

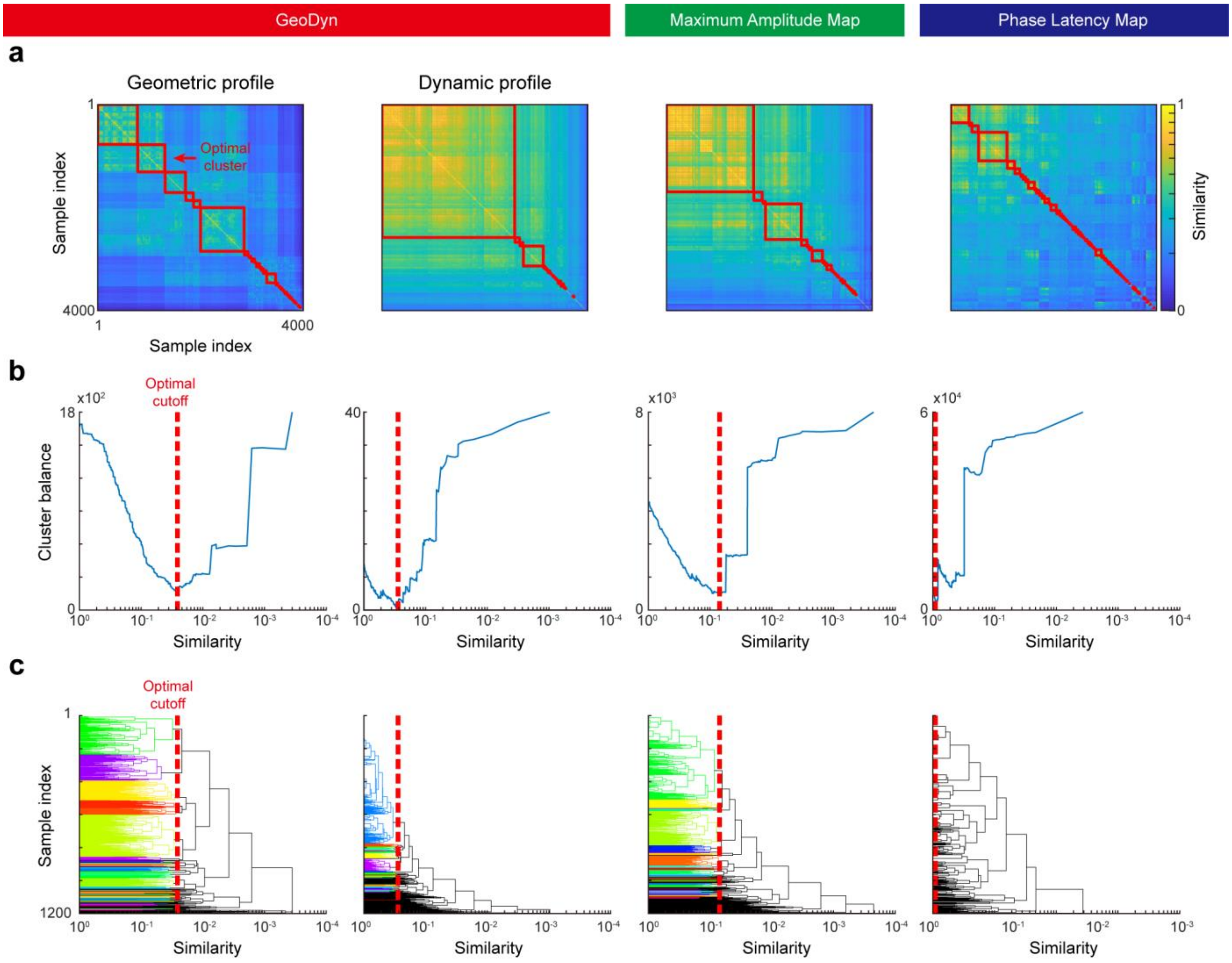

Supplementary Figure S7. Optimal clustering results of VSDI samples

(a) Similarity matrices between the geometric profiles, dynamic profiles, maximum amplitude map, and phase latency map of the sample VSDI data. Red squares indicate the samples in the same cluster. (**b)** The cluster balance was estimated for the optimal clustering of geometric profiles, dynamic profiles, maximum amplitude map, and phase latency map. The optimal cutoff value (red dash line) of clustering was set to minimize the cluster balance value. (**c)** Clustering results of the geometric profiles, dynamic profiles, maximum amplitude map, and phase latency map using optimal cutoff value.

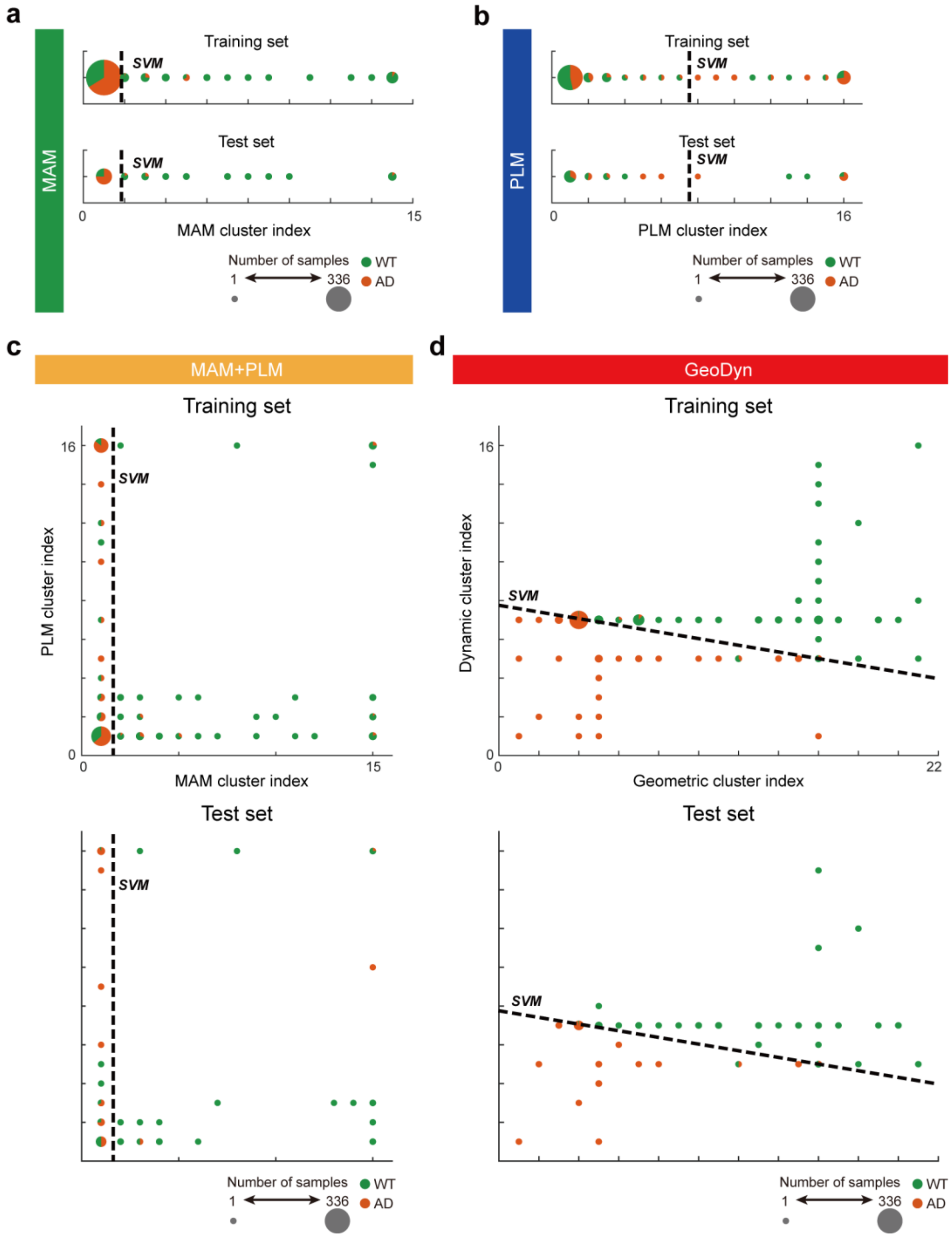

Supplementary Figure S8. Classification result of the WT and AD mice from VSDI data using MAM, PLM, the combination of MAM+PLM and GeoDyn. Each pie graph represents a ratio of the number of AD and WT samples belonging to each geometric and dynamic profile pair as orange and green colors, respectively. The size of a circle indicates the total number of samples grouped in a profile. The SVM linear classifier (black dash lines) was trained using the clustering results from training sets of data for **(a)** MAM, **(b)** PLM, **(c)** the combination of MAM+PLM, and **(d)** GeoDyn.

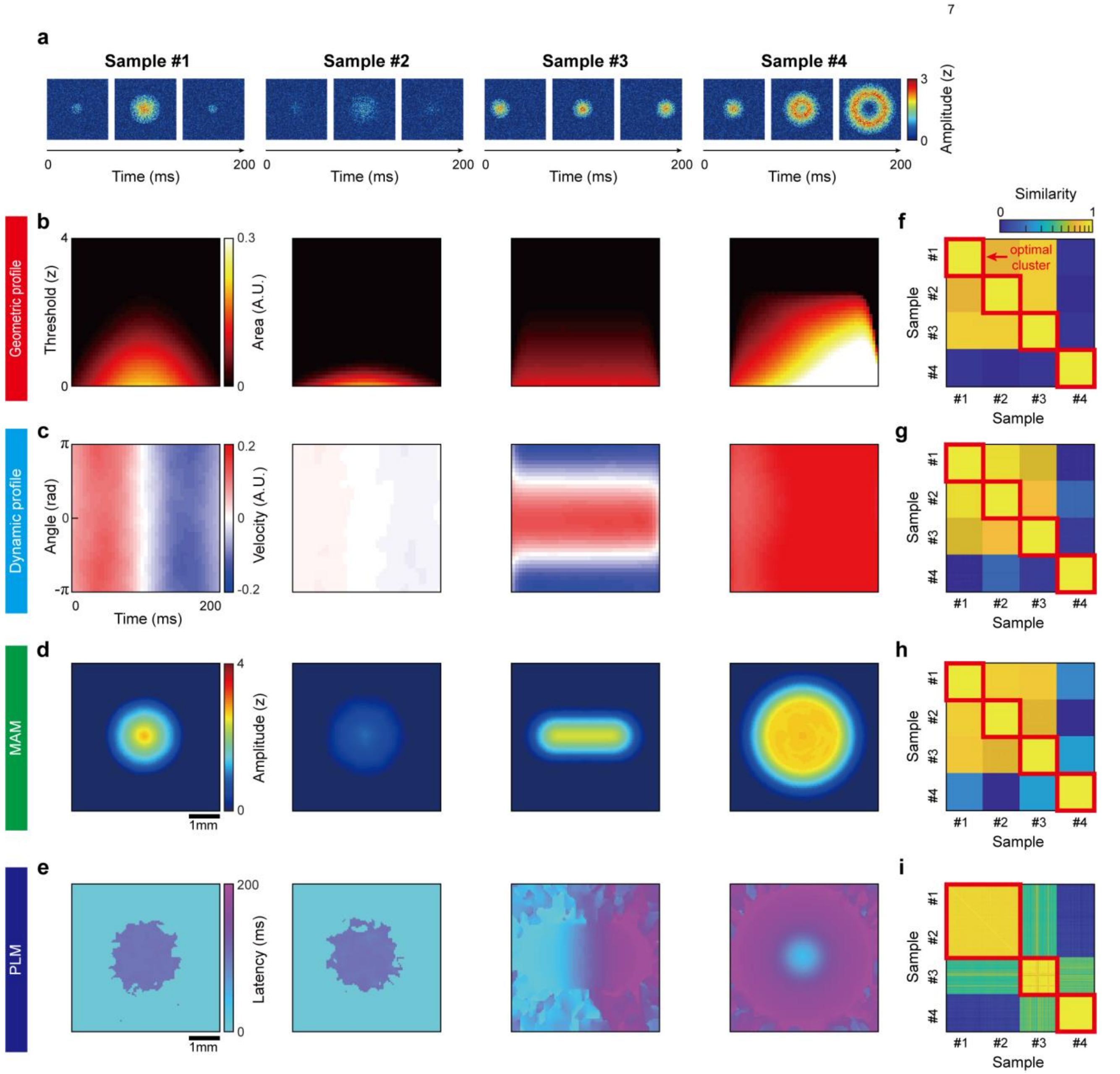

Supplementary Figure S9. Spatiotemporal pattern analysis with the Geometric profile, Dynamic profile, MAM, and PLM:

(a) Simulated samples of different spatiotemporal patterns. In Sample #1 and #2, the activity area increases and decreases over time, but keeps exactly the same size and peak location between the two. Sample #1 has slightly higher amplitude. The activity pattern in Sample #3 is moving linearly, and that in Sample #4 is propagating as a ring-shape. **(b-e)** The profiles of each sample activity. **(b)** Geometric and **(c)** Dynamic profiles. **(d)** MAM. **(e)** PLM. **(f-i)** Similarity matrices from **(f)** Geometric profile, **(g)** Dynamic profile, **(h)** MAM, and **(i)** PLM of the sample VSDI data. Red squares indicate the optimal cluster. Sample #1 and #2 were clustered together when PLM is used, while the other profiles well distinguish them.

Supplementary Video 1-3. Sample noisy activities of spatial pattern

1. Two stationary activities with noise

2. A stationary activity (left) and size variation (right)

3. A stationary activity (left) and amplitude variation (right)

Supplementary Videos 4–6. Sample noisy activities of dynamic motion

4. Two linearly moving activities with noise

5. A linearly moving activity (left) and speed variation (right)

6. A linearly moving activity (left) and spatial propagation (right)
